# Matrix metalloproteinase-1 induction by diethyldithiocarbamate is regulated via Akt and ERK/miR222/ETS-1 pathways in hepatic stellate cells

**DOI:** 10.1042/BSR20160111

**Published:** 2016-08-24

**Authors:** Tianhui Liu, Ping Wang, Min Cong, Dong Zhang, Lin Liu, Hongyi Li, Qingling Zhai, Zhuo Li, Jidong Jia, Hong You

**Affiliations:** * Liver Research Center, Beijing Friendship Hospital, Capital Medical University, Beijing Key Laboratory of Translational Medicine in Liver Cirrhosis & National Clinical Research Center of Digestive Disease, Beijing 100050, China

**Keywords:** diethyldithiocarbamate, ETS-1, liver fibrosis, matrix metalloproteinase-1, microRNA 222

## Abstract

Matrix metalloproteinase-1 (MMP-1) plays an important role in fibrolysis by degrading excessively deposited collagen I and III. We previously demonstrated that diethyldithiocarbamate (DDC) up-regulates MMP-1 in hepatic stellate cells via the ERK1/2 and Akt signalling pathways. In the current study, we attempted to further explore the molecular mechanisms involved in the regulation of MMP-1. We treated a co-cultured system that included hepatocytes (C3A) and hepatic stellate cells (LX-2) with DDC. The data revealed that the transcriptional factor ETS-1, which is an important regulator of MMP-1, was up-regulated in LX-2 cells following DDC treatment. Furthermore, the up-regulation of MMP-1 by DDC has been abrogated through employing si-ETS-1 to block expression of ETS-1. We found that DDC significantly inhibited the expression of miR-222 in LX-2 cells. We transfected miR-222 mimic into LX-2 cells and then co-cultured the cells with C3A. The up-regulation of ETS-1 and MMP-1 in LX-2 cells treated with DDC were inhibited after miR-222 mimic transfection. These data indicate that DDC up-regulated MMP-1 in LX-2 cells through the miR-222/ETS-1 pathway. Finally, we treated the co-cultured system with an Akt inhibitor (T3830) and an ERK1/2 inhibitor (U0126). Both T3830 and U0126 blocked the suppression of miR-222 by DDC in LX-2. Collectively, these data indicate that DDC up-regulated MMP-1 in LX-2 cells through the Akt and ERK/miR-222/ETS-1 pathways. Our study provides experimental data that will aid the control of the process of fibrolysis in liver fibrosis prevention and treatment.

## INTRODUCTION

Liver fibrosis results from continuous liver injury caused by viral hepatitis, alcoholic hepatitis, nonalcoholic steatohepatitis, drugs, metabolic diseases, autoimmune diseases and congenital abnormalities [1–2]. The end stage of liver fibrosis is cirrhosis, which is characterized by the appearance of regenerative nodules accompanied by hepatic failure and portal hypertension [3]. Cirrhosis represents an enormous worldwide healthcare problem. Increasing evidence suggests that advanced fibrosis and even cirrhosis are reversible [4–6]. Therefore, new and effective antifibrotic treatments for established liver fibrosis are earnestly required.

Liver fibrosis is characterized by the excessive depositions of types I and III collagen fibrils in the space of Disse [7]. Increased collagenolytic activity is a major mechanism of fibrosis resolution [8], and fibrillar collagens (type I and III) are degraded by interstitial matrix metalloproteinases (MMPs, MMP-1 in humans and MMP-13 in rats). According to several animal models of liver fibrosis resolution, interstitial MMPs continue to be expressed during resolution, which results in increased MMP activity and consequent matrix degradation within the liver [9–11]. Therefore, an improved understanding of the regulation of MMP-1/-13 activity in physiological and pathological conditions could lead to novel therapeutic interventions for liver fibrosis.

Diethyldithiocarbamate (DDC) is a major metabolite of disulfiram, which is known to be a potential agent for the treatment of alcoholism [12]. Many clinical aspects of DDC have been studied, including its use in treatments for metal toxicity, AIDS and cancers [13–17]. Additionally, DDC is a well-known NF-κB inhibitor. DDC interferes with the NF-κB pathway by inhibiting the nuclear translocation of NF-κB [18] and inhibiting IκB phosphorylation and proteasome degradation [19]. We previously demonstrated that DDC up-regulates MMP-1 in hepatic stellate cells via the ERK1/2 and Akt signalling pathways [20].

In the current study, we attempted to further explore the molecular mechanisms involved in the regulation of MMP-1. In this study, we used a co-culture model that is based on the co-incubation of a human hepatic cell line (C3A) with human hepatic stellate cells (LX-2) to examine the hypothesis that the up-regulation in MMP-1 by DDC involves miR-222 and its direct target gene, ETS-1, in LX-2 cells. Additionally, we investigated the roles of the Akt and ERK1/2 pathways in the regulation of miR-222 expression.

## MATERIALS AND METHODS

### Cell culture

**Figure 1:**
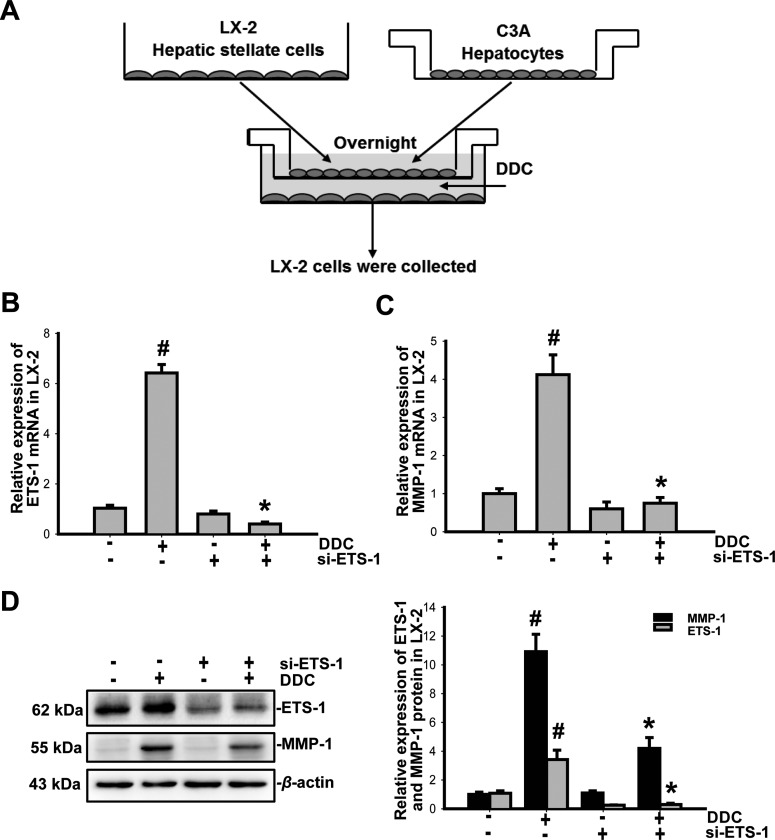
DDC up-regulates the expression of ETS-1 in LX-2 cells (**A**) Schematic of the co-culture model of C3A cells with LX-2 cells. LX-2 cells were transfected with si-ETS-1 (100 nM) and then co-cultured with C3A. The co-cultures were treated with 100 μM DDC, and LX-2 cells were collected and analysed at 24 h. (**B** and **C**) RT-PCR analysis of the total RNA to detect the ETS-1 and MMP-1 expression in LX-2 cells. (**D**) Fifty-microgram aliquots of the total protein extracts from LX-2 cells were subjected to immunoblot analysis with anti-human ETS-1 and MMP-1 antibodies and β-actin antibody as a loading control. The results were normalized according to β-actin and are expressed as fold increases relative to the control in each experiment. These experiments were repeated at least three times. The results are represented as the mean±the S.D. of three independent experiments. *P<0.05 compared with LX-2/C3A treated by DDC, # *P*<0.05 compared with the untreated LX-2/C3A cells.

The model used in the majority of the experiments described below was based on the co-culturing of the C3A cell line (ATCC® CRL-10741, the gift from Dr Yu Chen) with LX-2 cells (kindly provided by Dr Lieming Xu) (Figure 1A). The cells were co-cultured using cell culture inserts to separate the cell populations; LX-2 cells were plated on the bottom, and the C3A cells were plated on the insert to create a gravity gradient of the released mediators. A 5:1 ratio of C3A and LX-2 cells was selected because this ratio is representative of the ratio of parenchymal to nonparenchymal cells in the liver. After overnight incubation of LX-2 cells alone in DMEM supplemented with 10% fetal bovine serum, LX-2 medium was discarded, the cell culture inserts containing the overnight-incubated C3A cells were transferred, and the medium from these cells was added to the co-culture system. At this time, DDC (Sigma–Aldrich) and the ERK1/2 inhibitor U0126 (Promega) or the Akt inhibitor T3830 (Sigma–Aldrich) were added (*t*=0 h).

### si-ETS-1 and miR-222-3p mimic transfection

LX-2 cells were seeded in a six-well plate at a density of 4×10^5^ cells per well. Next, the medium was replaced with Opti-MEM, and the cells were transfected with si-ETS-1 (100 nM) or miR-222-3p mimic (100 nM) (RiboBio) and the negative control siRNA (100 nM) or miRNA (100 nM) using Lipofectamine 2000 transfection reagent (Invitrogen). After 6 h of transfection, the medium was replaced with DMEM containing 10% FBS and co-cultured with C3A. Then, after DDC treatment for 24 h, LX-2 cells were collected for total RNA and protein extraction.

### Quantitative miRNA PCR analysis

The total RNA was isolated from LX-2 cells using a miRcute miRNA kit (Tiangen) according to the manufacturer's instructions. To detect the miRNA expression, the RT reaction was performed using the miRcute miRNA cDNA kit (Tiangen). miRNA-222-3p and the U6 primers for the PCR reactions were obtained from Guangzhou RiboBio. Each sample was normalized according to the difference in the critical thresholds (CT) between miRNA-222-3p and U6 and relative to the control, and the amount of miRNA-222-3p was calculated as 2^−ΔΔCT^. All experiments were performed independently three times, and the averages were used for the comparisons.

### Quantitative RNA PCR analysis

The total RNA was isolated from LX-2 cells using TRIzol® reagent (Invitrogen) according to the manufacturer's instructions. The total RNA yields were quantified, and the equivalent amounts of total RNA (2 μg) were reverse-transcribed into single-stranded cDNA. Equal amounts of cDNA were subjected to PCR in the presence of SYBR green dye using the ABI Power SYBR Green PCR Master Mix kit (ABI Applied Biosystems) on an ABI Prism 7500 Sequence Detector (Applied Biosystems). PCR without a template was used as the negative control. *β-actin* mRNA was used as an internal control. The following primers were used: *MMP-1* forward: 5′-GATGAAGTCCGGTTTTTCAAAG-3′, reverse: 5′-GGGGTATCCGTGTAGCACCAT-3′; *ETS-1* forward: 5′-TGGAGTC AACCCAGCCTATC-3′; reverse: 5′-TCTGCAAGGTGTCTGTCTGG-3′ and *β-actin* forward: 5′-AGCAAGCAGGAGTATGACG-3′, reverse: 5′-AAAGGGTGTAACGCAACTAA-3′. PCR was performed with 45 cycles of 15 s at 95°C and 60 s at 60°C after a 2-min initial denaturation at 95°C. Each sample was normalized according to the difference in the critical thresholds (CT) between the target gene and *β-actin* and relative to the control. The amount of the target was calculated as 2^−ΔΔCT^. All experiments were performed independently three times, and the averages were used for the comparisons.

### Protein extraction and Western blot analysis

LX-2 cells were rinsed with PBS and immediately solubilized in lysis buffer at 4°C for 30 min. Following microcentrifugation at 14000 ***g*** for 5 min, the supernatants were transferred into a new tube, and the sample protein concentrations were determined using the Pierce Protein assay kit (Pierce). The protein mixtures were loaded into each well and separated on 12% SDS-PAGE electrophoresis gels. Following a 2-h run, the proteins were transferred onto nitrocellulose membranes (Amersham Biosciences). The membranes were blocked and subsequently incubated with anti-ETS-1 (Abcam), anti-MMP-1, anti-ERK1/2, anti-phospho-ERK1/2, anti-Akt, anti-phosphor-Akt (R&D Systems) and anti-β-actin (Sigma–Aldrich) antibodies at 4°C overnight. After extensive washing, the membranes were incubated with the secondary antibody for 60 min followed by extensive washes. Specific antibody–antigen complexes were detected with ECL Western blot detection kits (Pierce). All experiments were performed independently three times, and the averages were used for the comparisons. Protein expression was quantified via densitometric analyses of the immunoblots using the Quantity One software.

### Statistical analysis

All values are indicated as the mean±the standard deviations of the mean (SEMs). Two-group comparisons were performed with Student's *t* tests. Comparisons of the mean values of three or more groups were performed with ANOVAs. Differences of *P*< 0.05 were considered statistically significant.

## RESULTS

### DDC up-regulates ETS-1 expression in LX-2 cells

Previous studies have demonstrated that MMP-1 expression is regulated by ETS-1. However, the regulatory role of ETS-1 in the mediation of the transcriptional activity of MMP-1 is likely cell- and tissue-specific [21]. To determine whether ETS-1 was involved in the regulation of MMP-1 in LX-2 cells by DDC, we treated the co-cultures with 100 μM DDC for 24 h. LX-2 cells were collected, and ETS-1 and MMP-1 expression were analysed with real-time PCR and Western blot. Compared with the control without DDC, the expression of both the *ETS-1* (1.03±0.12 compared with 6.42±0.34, *P*<0.05) and *MMP-1* (1.00±0.13 compared with 4.12±0.52, *P*<0.05) mRNAs in LX-2 cells were simultaneously significantly increased following treatment with DDC (Figures 1B and 1C). Compared with LX-2 cells that were treated with DDC, the si-ETS-1 inhibited the DDC-mediated up-regulation of *ETS-1* (6.42±0.34 compared with 0.4±0.08, *P*<0.05) and *MMP-1* (4.12±0.52 compared with 0.75±0.15, *P*<0.05) mRNAs in LX-2 cells (Figures 1B and 1C).

Compared with the control, the expression of both the ETS-1(1.08±0.17 compared with 3.42±0.65, *P*<0.05) and MMP-1 (1.00±0.16 compared with 10.92±1.21, *P*<0.05) protein in LX-2 cells were significantly increased following treatment with DDC (Figure 1D). Compared with LX-2 cells that were treated with DDC, the si-ETS-1 inhibited the DDC-mediated up-regulation of ETS-1 (3.42±0.65 compared with 0.47±0.08, *P*<0.05) and MMP-1 (10.92±1.21 compared with 4.20±0.75, *P*<0.05) in LX-2 cells (Figure 1D). These data suggest that the up-regulation of MMP-1 by DDC was associated with ETS-1.

### DDC inhibits miR-222-3p expression in LX-2 cells

**Figure 2:**
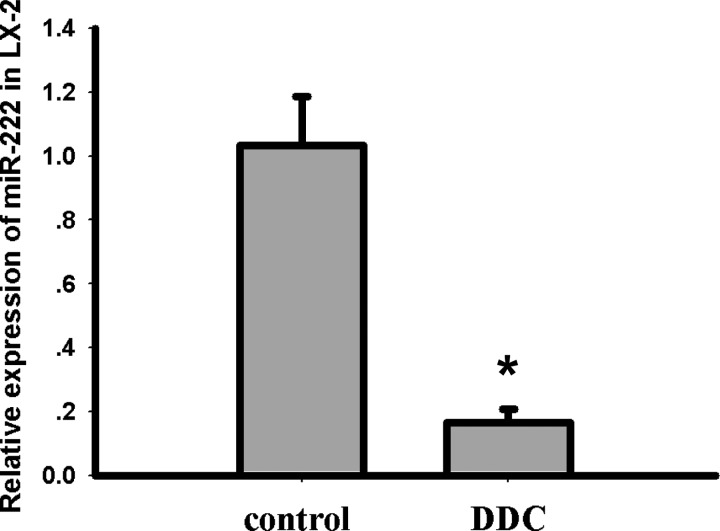
DDC inhibits the expression of miR-222 in LX-2 cells The co-culture systems were treated with 100 μM DDC for 24 h. LX-2 cells were collected and analysed for miR-222 expression by RT-PCR. The results were normalized to U6 and are expressed as fold increases relative to the control in each experiment. These experiments were repeated at least three times. The results are represented as the mean±the S.D.s of three independent experiments. * *P*< 0.05 compared with the untreated LX-2/C3A cells.

DDC is a potent inhibitor of NF-κB [22,23], which induces the expression of miR-222 by binding with the promoter of miR-222 [24]. After demonstrating that DDC up-regulated the expression of ETS-1, we investigated whether DDC regulated miR-222-3p, which directly targets ETS-1 and inhibits the expression of ETS-1. To this end, we treated the co-cultures with 100 μM DDC for 24 h. Following this treatment, LX-2 cells were collected and analysed for miR-222-3p. As illustrated in Figure 2, the miR-222-3p expression in LX-2 cells (0.17±0.04, *P*<0.05) was significantly inhibited in the presence of 100 μM DDC compared with the untreated LX-2 cells (1.03±0.15) that were co-cultured with the C3A cells. These data suggest that DDC inhibited the expression of miR-222-3p in LX-2 cells.

### DDC up-regulates MMP-1 by inhibiting miR-222/ETS-1

**Figure 3:**
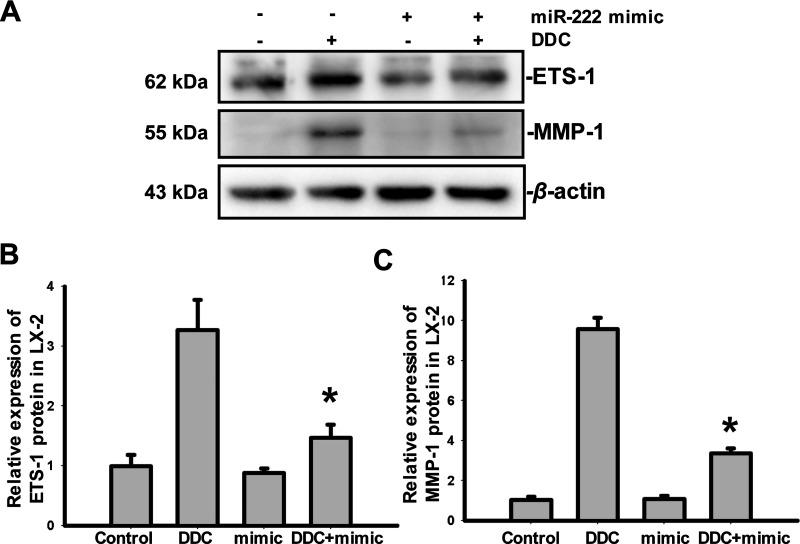
DDC up-regulates MMP-1 expression in LX-2 cells by inhibiting miR-222/ETS-1 LX-2 cells were transfected with miR-222-3p mimic (100 nM) and then co-cultured with C3A. The co-culture systems were treated with or without 100 μM DDC for 24 h. LX-2 cells were collected and analysed. (**A**) Fifty-microgram aliquots of total protein extracts from LX-2 cells were subjected to immunoblot analysis with anti-human ETS-1 and MMP-1 antibodies and β-actin antibody as a loading control as described in the Materials and Methods section. (**B** and **C**) The relative expression of ETS-1 and MMP-1. These experiments were repeated at least three times. The results are representative of the mean±the S.D.s of three independent experiments. * *P*<0.05 compared with LX-2/C3A treated with DDC.

Because DDC up-regulates ETS-1 and MMP-1 while inhibiting miR-222-3p in LX-2 cells, we next attempted to determine whether ETS-1 and MMP-1 are the downstream targets of miR-222-3p. To this end, we transfected LX-2 cells with a miR-222-3p mimic (100 nM) to overexpress miR-222-3p. As illustrated in Figure 3, compared with LX-2 cells that were treated with DDC, the miR-222-3p mimic blocked the DDC-mediated up-regulation of both ETS-1 (3.27±0.51 compared with 1.47±0.22, *P*<0.05) and MMP-1 (9.57±0.57 compared with 3.37±0.25, *P*<0.05) in LX-2 cells. However, compared with the control, the miR-222-3p mimic transfection did not influence the expression of ETS-1 (0.99±0.19 compared with 0.88±0.07, *P* > 0.05) or MMP-1 (1.03±0.15 compared with 1.08±0.12, *P* > 0.05) in LX-2 cells that did not undergo DDC treatment. These data indicate that DDC inhibited miR-222-3p and subsequently enhanced the ETS-1/MMP-1 pathways.

### DDC inhibits miR-222 through the Akt and ERK1/2 pathways

**Figure 4:**
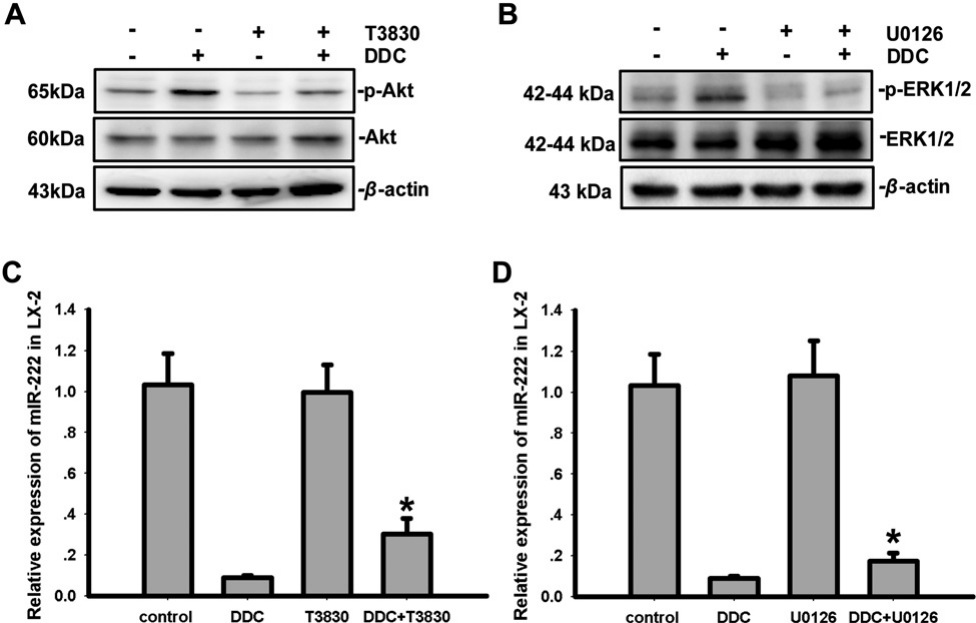
DDC inhibits the expression of miR-222 in LX-2 cells through Akt and ERK1/2 pathways (**A**) Co-cultures were treated with or without 100 μM DDC for 1 h in the presence or absence of an Akt inhibitor (T3830, 50 μM). The phosphorylation levels of Akt were analysed by Western blotting. (**B**) Co-cultures were treated with or without 100 μM DDC for 1 h in the presence or absence of an ERK1/2 inhibitor (U0126, 10 μM). The phosphorylation levels of ERK1/2 were analysed by Western blotting. (**C**) Co-cultures were treated with or without 100 μM DDC for 24 h in the presence or absence of an Akt inhibitor (T3830, 50 μM). The MMP-1 protein levels were analysed by Western blotting. (**D**) The co-cultures were treated with or without 100 μM DDC for 24 h in the presence or absence of an ERK1/2 inhibitor (U0126, 10 μM). The MMP-1 protein levels were analysed by Western blotting. These experiments were repeated at least three times. The results are represented as the mean±the S.D.s of three independent experiments. * *P*< 0.05 compared with LX-2/C3A treated by DDC.

We previously demonstrated that DDC up-regulates MMP-1 in hepatic stellate cells via the ERK1/2 and Akt signalling pathways [20]. Here, we attempted to investigate whether the Akt and ERK1/2 signalling pathways were involved in the regulation and control of miR-222 by DDC. We treated the co-cultures with or without 100 μM DDC for 1h in the presence or absence of an Akt inhibitor (T3830, 50 μM) or an ERK1/2 inhibitor (U0126, 10 μM). As shown in Figures 4(A) and 4(B), DDC activated the phosphorylation of Akt or ERK1/2, and T3830 and U0126 effectively inhibited the phosphorylation of Akt or ERK1/2 in LX-2 cells induced by DDC respectively.

We treated the co-cultures with or without 100 μM DDC for 24 h in the presence or absence of an Akt inhibitor (T3830, 50 μM) or an ERK1/2 inhibitor (U0126, 10 μM). As illustrated in Figures 4(C) and 4(D), compared with LX-2 cells that were treated with DDC, both T3830 (0.09±0.01 compared with 0.30±0.08, *P*<0.05) and U0126 (0.09±0.01 compared with 0.18±0.38, *P*<0.05) blocked the inhibition of miR-222 by DDC in LX-2 cells. Moreover, compared with the control, T3830 (1.01±0.14 compared with 0.99±0.14, *P* > 0.05) and U0126 (1.01±0.14 compared with 1.08±0.17, *P* > 0.05) did not influence the expression of miR-222 in LX-2 in the cells that were not treated with DDC. These results indicate that the inhibition of miR-222 by DDC was Akt- and ERK1/2-dependent.

**Figure 5:**
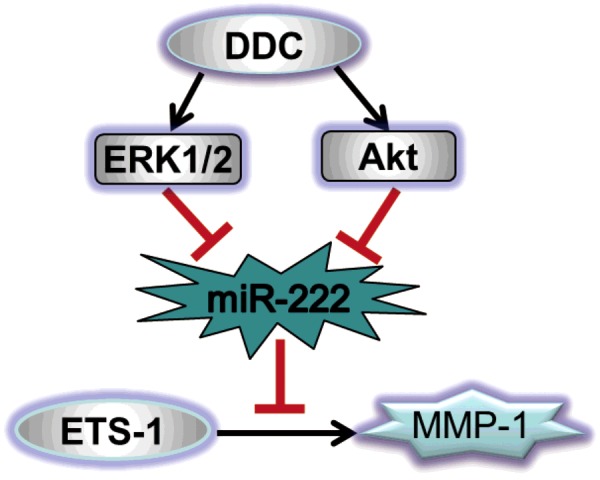
Signalling pathways involved in the regulation of MMP-1 by DDC in LX-2 cells

Collectively, these results indicate that DDC up-regulates MMP-1 in LX-2 cells through the Akt and ERK/miR-222/ETS-1 pathways (Figure 5).

## DISCUSSION

MMP-1 gene expression is stimulated at the transcriptional level by various cytokines, growth factors and tumour promoters via a promoter segment, which contains adjacent binding sites for ETS and AP-1 transcription factors [25–27]. Because DDC induces MMP-1 expression in LX-2 cells [20], it is important to note the role of ETS-1 in the DDC-induced up-regulation of MMP-1. In the present study, we found that both the mRNA and protein of ETS-1 in LX-2 cells significantly increased when treated with DDC, and these increases were accompanied by an up-regulation of MMP-1. Meanwhile, the up-regulation of MMP-1 by DDC can be abrogated through employing a specific siRNA to block expression of ETS-1. These data suggest that ETS-1 plays a role in the up-regulation of MMP-1 by DDC.

miRNAs are short 20–22 nucleotides that act as negative regulators of gene expression by inhibiting protein translation or inducing mRNA degradation [28]. It has been reported that ETS-1 is the direct target of miR-222 in cardiomyocytes and melanocytes. The transfections of miR-222 in the presence of ETS-1 3’UTR induce a significant decrease in luciferase activity [29]. Here, we investigated whether miR-222 was involved in the regulation of ETS-1 by DDC. Our data revealed that DDC significantly inhibited the expression of miR-222 in LX-2 cells. After the overexpression of miR-222 in LX-2 cells through the transfection of miR-222 mimic, both the increases in ETS-1 and MMP-1 by DDC were blocked. These data indicated that the inhibition of miR-222 by DDC mediated the up-regulation of ETS-1 and the subsequent increase in MMP-1 in LX-2 cells.

Mitogen-activated protein kinases (MAPKs) are a ubiquitous group of serine/threonine kinases, that play a crucial role in transmitting the transmembrane signals required for cell growth, differentiation and apoptosis. Previous studies have demonstrated that the MAPK pathways mediate the MMP-1 expression induced by various stimuli [30,31]. For example, the ERK1/2 pathway mediates the activation of the MMP-1 promoter via an AP-1 element induced by Ras, serum, phorbol ester, insulin, and the specific activation of ERK1/2 induces MMP-1 production [32,33]. We previously reported that DDC activated ERK1/2 and then mediated the up-regulation of MMP-1 in LX-2 cells [20]. In the present study, we found that the ERK1/2 inhibitor U0126 suppressed the inhibition of miR-222 by DDC. These data indicate that ERK1/2 mediated the up-regulation of MMP-1 by DDC by inhibiting the miR-222/ETS-1 pathways.

Akt is a serine/threonine protein kinase that plays a critical role in controlling the balance between apoptosis and cell survival in response to extra- and intracellular signalling [34]. Previous studies have demonstrated that Akt activation is also involved in MMP-1 secretion [35,36]. In the present study, we found that the Akt inhibitor T3830 suppressed the inhibition of miR-222 by DDC. These data indicate that Akt also mediated the up-regulation of MMP-1 by DDC by inhibiting miR-222/ETS-1 pathways.

In conclusion, as illustrated in Figure 5, our studies revealed a novel mechanism for MMP-1 regulation via the Akt and ERK/miR-222/ETS-1 pathways. We believe that these results will provide important theoretical and experimental bases needed to control interstitial MMPs (MMP-1 in humans and MMP-13 in rats) and lead to the resolution of liver fibrosis. Additionally, it is possible that the inhibition of the effects of miR-222 could be a useful future therapeutic goal for liver fibrosis.

## Abbreviations

Akt: protein kinase B
DDC: diethyldithiocarbamate
ERK: extracellular signal-regulated kinase
ETS-1: E26 transformation-specific-1
MAPK: mitogen-activated protein kinases
MMP-1: matrix metalloproteinase-1
